# The Characteristics of Autistic Children Attending a Neuro-Developmental Center in Northern Sri Lanka

**DOI:** 10.7759/cureus.35970

**Published:** 2023-03-10

**Authors:** Bhavana Sivayokan, Sambasivamoorthy Sivayokan, Kumanan Thirunavukarasu, Gitanjali Sathiadas, Tharmila Sivapathamoorthy

**Affiliations:** 1 Community and Family Medicine, Faculty of Medicine, University of Jaffna, Jaffna, LKA; 2 Mental Health Unit, Teaching Hospital Jaffna, Jaffna, LKA; 3 Psychiatry, Faculty of Medicine, University of Jaffna, Jaffna, LKA; 4 Medicine, Faculty of Medicine, University of Jaffna, Jaffna, LKA; 5 Paediatrics, Faculty of Medicine, University of Jaffna, Jaffna, LKA

**Keywords:** developmental psychology, child development, early detection, early intervention, autism spectrum disorder (asd)

## Abstract

Introduction: The global prevalence of autism spectrum disorder is rising. However, the services related to autism are only slowly being developed in poor-resource nations. There is a need to understand the characteristics of autistic children in order to develop individualized, evidence-based interventions. This study aims to analyze the sociodemographic profile and initial clinical presentations of autistic children in the northern part of Sri Lanka and determine the differences in these factors between this region and the rest of the country and the globe.

Methods: This retrospective descriptive study in a center for neuro-developmental disorders in Northern Sri Lanka analyzed data extracted from clinical records of 123 autistic children using a predesigned data extraction form. Descriptive analyses and chi-square tests were performed using RStudio.

Results: Among the 123 children, 71.5% were males. The mean age of diagnosis was 3.4 years. Most children (69.9%) had mild to moderate symptoms of autism. At the time of presentation, all children had speech-related complaints, while behavioral issues, poor social interaction, and sensory issues were reported in 91.9%, 96.7%, and 78% respectively. Social stigma was found to be the prime challenge faced by caregivers.

Conclusion: The findings show that speech and language-related problems are universal among autistic children in this region, while behavioral issues, poor social interaction, and sensory issues are highly prevalent. Further, this study highlights the need for community awareness through the primary healthcare system to address delays in detecting red-flag signs of autism by parents and seeking professional help.

## Introduction

The global prevalence of autism spectrum disorder (ASD) has increased from 0.62% in 2012 to 1.0% in 2021 [1]. In par with the global tendency, the prevalence of ASD is on the rise in Sri Lanka. The only study that explored the prevalence of autism in Sri Lanka [2] found that red-flag signs of autism, as specified by the American Academy of Neurology and Child Neurology Society, were present in 7.4% and that one in 93 children (1.07%) aged 18 to 14 months was diagnosed with autism. Although this prevalence was estimated over a decade ago, it is higher than the prevalence in other parts of South Asia [3] and closely resembles the current global prevalence.

Interventions for autistic children consume a lot of resources and facilities, especially due to the necessity of having a larger therapist/client ratio as each autistic child needs an individualized set of interventions. In Sri Lanka, facilities that provide such care have been set up in the government and private sectors predominantly in and around the capital of the island nation. In this background, a center for neuro-developmental disorders, Mathavam, was developed in 2014 in Jaffna, which has a population of 616,462 [4], with a public-charity partnership. Though this center was established to fulfill the need of this region, children from other parts of the country, as well as children of expatriates, also benefitted immensely from this center over the years.

At the time of study, more than six years had elapsed since this center’s inception, and it was found pertinent to explore the characteristics of the children this center serves. This study was carried out to analyze the sociodemographic profile of the children and their families, and the clinical presentation at the time of initial contact, and determine the differences in these factors between this region and the rest of the country and the globe, as it is crucial to improve services and provide tailor-made care for these children in the future.

## Materials and methods

Study design and duration

This retrospective descriptive study on a cohort was conducted in Mathavam, a center for neuro-developmental disorders in Jaffna, Sri Lanka. Details of all the children who were diagnosed with ASD, according to the Diagnostic and Statistical Manual of Mental Disorders 5 criteria, and assessed for severity of autism, using the Childhood Autism Rating Scale (CARS), at Mathavam from 2017 to 2020 were obtained from the existing records at the center.

Data extraction

Data extraction was carried out by members of the research team during the early part of 2021 using a pre-designed electronic data extraction form. No exclusion criteria were applied. The details of the children extracted included the child’s gender, place of birth, mode of delivery, early neonatal complications, and details pertaining to suspecting autism in the child and the diagnosis of ASD. Details of the parents, including consanguinity in their marriage, as well as family history of autism and other neurodevelopment disorders were also obtained.

Extracted details of the clinical presentation included chief presenting complaints, CARS score at initial assessment and the severity based on this score, and comorbid conditions. After going through the literature, the chief presenting complaints were analyzed under five categories: speech-related issues, social interaction-related issues, repetitive behaviors, other behavioral problems, and sensory issues. The selected speech-related issues include no speech, uttering only a few words, and speech regression, social interaction-related issues include poor eye contact, poor name response, and poor interaction with others, repetitive behaviors include stereotyped body movements and stereotyped play activities, other behavioral issues include hyperactivity, temper tantrums, self-injurious behaviors, and physical aggression, and sensory issues include hyporeactivity and hyperreactivity to visual, auditory, tactile, gustatory, and olfactory stimuli as well as spatial orientation and pain.

In addition to these details, the challenges faced by the parents as well as the therapists during the course of providing intervention were also extracted from the clinical notes. Challenges perceived by the parents were grouped as issues with time commitment for therapy, traveling difficulties, economic difficulties, social stigma, poor family support, and struggles in taking care of other children and family members. The challenges perceived by the therapists were grouped as poor attendance of the parents at the therapy sessions, which is defined as being absent continuously for more than a week, poor compliance of parents, which is defined as not following the therapists’ instructions in a systematic manner at home, poor understanding of the parents, unrealistic expectations of the parents, and demanding nature of the parents.

Data analysis

Data analysis was carried out using the software RStudio, and included basic descriptive statistics, such as percentage, mean, median, mode, minimum, maximum, and standard deviation. Association between the challenges faced by the therapists and parents, and the educational level of the parents was analyzed using chi-square test.

Community involvement

This study was co-led by members of the autism community.

Ethical considerations

Ethical clearance for this study was granted by the Ethics Review Committee of the Faculty of Medicine, University of Jaffna on February 18, 2021 (Reference number: J/ERC/20/120/NDR/0240). Since this study utilized secondary data, individual patient consent was waived off.

## Results

The details of 123 children diagnosed with autism were obtained from the records at this center. The key sociodemographic details are summarized in Table 1.

**Table 1:** Sociodemographic characteristics of the children (N=123)

Characteristics	Children (N=123)	
	no.	%
Gender		
Male	88	71.5
Female	35	28.5
Child's position in the family		
First-born	95	77.2
Second-born	23	18.7
Third-born	5	4.1
Place of birth		
Sri Lanka	91	74.0
Other country	32	26.0
Mode of delivery		
Normal vaginal delivery	49	39.8
Assisted vaginal delivery	5	4.1
Cesarean section	69	56.1
Neonatal complication		
No complication	98	79.7
Had complication	25	20.3

It was found that the mean age at which the condition was suspected was 2.7 years (SD 0.89). In most children (60.2%, n=74), the condition was first suspected by the mother, while a healthcare worker suspected autism in 12 (9.8%) children. The mean age at which autism was diagnosed was 3.4 years (SD 1.04). A diagnosis of ASD was made by a pediatrician in 47 (38.2%) children and a psychiatrist in 44 (35.8%) children.

The mean age at which initial help was sought from Mathavam was found to be 3.7 years (SD 1.67). Among the 123 children, 43 (35.0%) were referred to Mathavam by the psychiatrist, 42 (34.1%) by the pediatrician, 11 (8.9%) by other institutions, and three (2.4%) by preschool teachers. Further, 21 (17.1%) children were brought by their parents on their own. Nearly three-fourths of the families (73.9%, n=91) first heard of this institution from their doctors and other healthcare workers, while 18 families (14.4%) had heard about it from friends.

The mean age of the mothers during the birth of the child was 29.7 years (SD 4.27), and that of the fathers was 34.0 years (SD 4.55). All the mothers and fathers had completed their education at least up to secondary education, with 49 (39.8%) mothers and 53 (43.1%) fathers having completed tertiary education. However, nearly two-thirds of the mothers (65.0%, n=80) were unemployed, whereas all the fathers were employed. Of the employed mothers, 42 (34.1%) were skilled workers or professionals, with only one (0.8%) mother being a semi-skilled worker. Among the fathers, 85 (69.1%) were skilled workers or professionals, while 38 (30.9%) were semi-skilled workers.

Considering the family history of these autistic children, two (1.6%) had first-degree relatives with ASD, 18 (14.6%) had second-degree relatives with ASD, and 11 (8.9%) had second-degree relatives with a neurodevelopmental disorder other than ASD.

**Figure 1:**
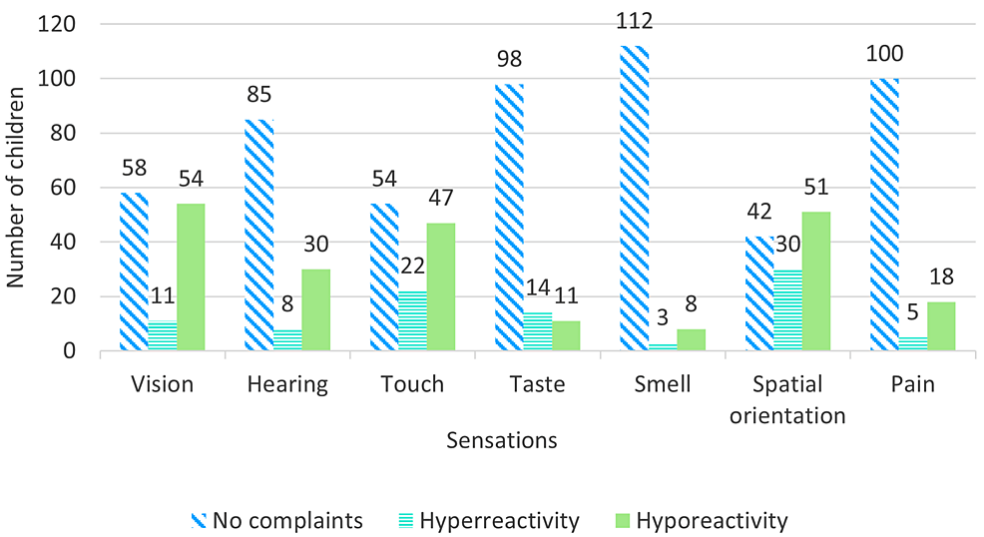
Prevalence of sensory issues among children with autism spectrum disorder (N=123)

When the clinical presentation of the children was considered, all the children had speech-related issues. Except for three (2.4%) children, all had complaints regarding some aspects of social interaction. Some form of sensory issue was found in 78% of the children with many having issues with spatial orientation, touch, and vision. The prevalence of sensory issues is shown in Figure 1. Repetitive behaviors were reported in 100 (81.3%) children. Among other behavioral problems, the most commonest was temper tantrums, which were found in 80 (65.0%) children. The important clinical features with which these children presented have been summarized in Table 2.

**Table 2:** Clinical presentation of the children (N=123)

Characteristics	Children (N=123)	
	no.	%
Speech related		
No speech	58	47.2
Few words only	59	48.0
Speech regression	6	4.9
Social interaction		
Poor eye contact	108	87.8
Not responding to call	117	95.1
Not interacting with others	119	96.7
Behavioral problems		
Repetitive behavior		
Stereotyped body movements	7	5.7
Stereotyped play activities	37	30.1
Both	56	45.5
Hyperactivity	46	37.4
Temper tantrum	80	65.0
Self-harming behavior	15	12.2
Physical aggression	16	13.0

**Figure 2:**
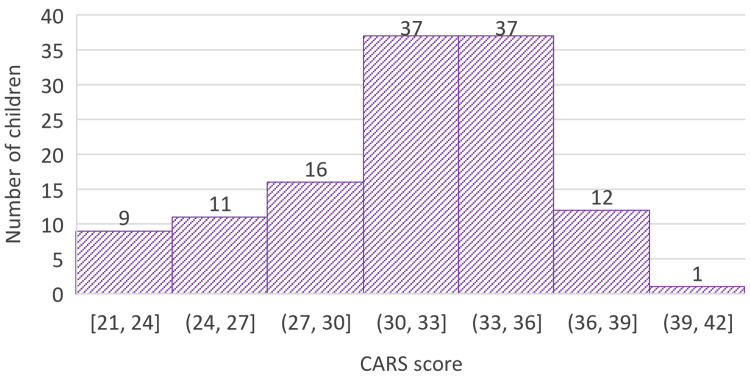
Childhood Autism Rating Scale (CARS) scores at initial assessment (N=123)

During the initial assessment at the center, CARS was used by the clinicians to assess the severity of autism. The possible CARS scores range from a minimum of 15 to a maximum of 60 [5]. The mean CARS score among the children assessed at this center was 31.9 (SD 4.2). The minimum CARS score obtained was 21, and the maximum was 40. The distribution of CARS scores of all 123 children included in this study is depicted in Figure 2. According to the CARS score, 27 (22.0%) children had minimal autism, 86 (69.9%) children were found to have mild-moderate autism, and 10 children (8.1%) were found to have severe autism. Among co-morbidities, eight (6.5%) children had mental retardation, while seven (5.7%) had attention deficit hyperactivity disorder (ADHD), five (4.1%) had epilepsy, and one (0.8%) had bronchial asthma.

It was found that mothers played the role of the primary caregiver for 121 children (98.4%), with a grandparent taking up that role for one (0.8%) child, and an extended family member for another. When the key challenges perceived by the caregivers were analyzed, it was found that the issue faced by many of the caregivers (78.9%, n=97) was social stigma. Further, 89 (72.4%) caregivers reported that they had poor family support. The time commitment for therapy was an issue for 65 (52.8%) caregivers. Further, 56 (45.5%) caregivers had traveling difficulties and 32 (26.0%) had economic difficulties, which hindered their commitment to therapy. In addition, 45 (36.6%) caregivers reported that they were struggling to take care of the other children or family members. Among these challenges, the economic difficulties of the parents showed statistically significant association with the educational level of the mothers $X^{2}(4, N=123)=12.354, p=.015$ and fathers $X^{2}(4, N=123)=14.976, p=.005$, with the parents having had a tertiary education facing less economic difficulties than the others.

The challenges perceived by the therapists at this center were also extracted from the notes in the clinical records. The therapists felt that poor attendance at the sessions was an issue for 56 (45.5%) children, poor compliance of the parents in matters related to the therapy for 43 (35.0%) children, and poor understanding of the parents for 29 (23.6%) children. The therapists felt that the parents of 13 (10.6%) children had unrealistic expectations, and the parents of 12 (9.8%) children were overly demanding. None of these challenges perceived by the therapists showed statistically significant associations with the education level of the parents. However, the poor attendance at the sessions was found to have a statistically significant association with the traveling $X^{2}(4, N=123)=125.47, p&lt; .001$ difficulties and the economic difficulties $X^{2}(4, N=123)=126.20, p&lt; .001$ of the parents.

## Discussion

The male preponderance of autistic children in this study tallies with the results obtained in a previous study conducted in Sri Lanka [6], and with the established male-to-female ratio, nearly 3:1, in the prevalence of ASD as stated in the literature [7].

Among the children included in this study, most (77.2%) were first-born. This preponderance of first-born children with a diagnosis of ASD was also noted in a study conducted in Bangladesh [8], although the percentage was lower, at 59.7%. The study conducted in Bangladesh further states that 9.1% of the children were born of consanguineous marriage, whereas this value was found to be 5.7% in this study.

The mean age of the mother and father at the time of birth of the child was found to be 29.7 and 34.0 years, respectively. This is similar to 31.6 and 34.5 years, which is described in a study conducted in the United States [9]. Nearly four-fifths of the children did not have any complications at birth. A similar result was obtained from a study carried out in Malaysia [10], in which it was reported that 85.8% of the children were term babies with normal birth weight and without complications. But, another study [11] reported higher perinatal complications among autistic children in Egypt (35.0%) and Saudi Arabia (42.8%).

The mean age at which autism was suspected was 2.7 years (32.4 months). However, the mean age of diagnosis was 3.4 years (40.8 months). This clearly reflects a delay in the establishment of a diagnosis despite the country’s health system, in which the maternal mortality rate, infant mortality rate, and vaccine coverage are comparable to the developed nations [12], and which permits easy and immediate access to specialists. This might be due to the unwillingness of the parents to seek medical attention due to stigma or denial, or a higher threshold on the healthcare provider’s part for diagnosing autism. This mean age of diagnosis is slightly higher than the 35.8 months that was found in the previous study in Sri Lanka [6]. The mean age of diagnosis, as revealed by this study is much lower when compared to the findings from studies in the other parts of South and Southeast Asia, where the mean age of presentation was much higher at 5.5 years [10], 6.5 years [13], and 6.6 years [8]. However, it is higher when compared to a study carried out in Egypt and Saudi Arabia [11], in which the mean age of presentation was 1.7 years in Saudi Arabia and 2.25 years in Egypt. In addition, the onset of treatment in these regions was also found to be at a lower mean age of 2.5 years. It is well documented that a delay in detection and the consequent delay in intervention would have unfavorable outcome [14]. Unawareness of autism among parents is a major contributory factor for these delays as inferred from the results of this study and supported by the results of a previous study [15]. Considering the findings from another study showing that over four-fifths of the public health midwives in this region have good knowledge of autism and its red-flag signs [16], utilizing them to provide community education could be a cornerstone in preventing such delays.

The mean age at which initial help was sought from this institution was found to be 3.7 years (44.4 months), which is higher than the values in Egypt and Saudi Arabia [11]. Nevertheless, it shows that even though there is a delay between the suspicion of the condition and the diagnosis, there is not much delay in seeking interventions after diagnosis. Coupled with the findings which state that most of the parents were referred to Mathavam by their doctors and that most parents got to know about this institution through a healthcare worker, it can be surmised that there is awareness among the healthcare professionals regarding the need to seek intervention as early as possible.

When considering the characteristics of the parents, it was found that while all the parents had at least some form of secondary education, nearly two-thirds of the mothers were unemployed. However, for all but two children included in this study, it was the mothers who took on the monumental task of being the primary caregiver for the autistic child. A deeper look into the education level of the parents shows that well over one-third of them (39.8% of mothers and 43.1% of fathers) have completed tertiary education. This finding is similar to that obtained in a study conducted in Bangladesh [17], which found one-third of the parents to be degree holders, and another study carried out in Malaysia [10], in which 38.7% of the fathers and 41.1% of the mothers were found to be degree holders. However, a larger proportion of parents with tertiary education was reported in a study carried out in the US [9] (83.6% of the mothers and 72.7% of fathers), and another study in Bangladesh [8] (56.5% of the mothers and 75.4% of the fathers). Most of the fathers being employed while most mothers were unemployed was also reported by Hussein and her team in their study among autistic children in Egypt and Saudi Arabia [11].

The CARS score during the initial assessment revealed that most children (69.9%) had mild-moderate autism, with only 8.1% having severe autism. Among the co-morbidities identified in this study, mental retardation was the one with the highest prevalence (6.5%), followed by ADHD (5.7%), and epilepsy (4.1%). This is contrary to previous studies conducted in Sri Lanka, which stated that epilepsy is among the most common co-morbidities in autistic children, with it being prevalent in 10.6% according to one study [6], and 19.8% according to another [18]. A study conducted in India [13] revealed that ADHD and epilepsy were the commonest co-morbidities. A Malaysian study [10] also concluded thus, stating that ADHD was found in 14.2% of the children and epilepsy in 9.4%. Not many studies have explored the prevalence of mental retardation in autistic children.

Several studies around the globe have analyzed the presenting complaints of autistic children. This study found that all the children who presented to Mathavam for intervention had some form of difficulty in speech, the predominant of which were no speech and limited speech. Other studies previously carried out in Sri Lanka have also recorded poor speech as one of the major complaints, with one study [6] claiming that 82.3% of the children had poor speech, while another study stated that 23.4% of the children had poor development of speech [18]. Almost all the autistic children were found to have language problems, according to the study on autistic children in Egypt [11]. In Italy, it was found that 92.4% of autistic children had language problems at the time of initial presentation [19]. Speech regression was found only in 4.9% of the children who presented to Mathavam. However, this value was almost three folds (14.8%) in a Malaysian study [10]. The first study that explores the presentation of children with autism in Sri Lanka reported that as much as 47.3% of the children had speech regression [6].

Although atypical sensory-related behaviors have been reported in over 90% of the children with autism [20], not many studies have analyzed in detail the sensory issues that autistic children from this region have. Nearly two-thirds of the children (65.8%) had abnormal spatial orientation, which can explain their problems with balance, and the need for modified behaviors to regulate themselves. However, it might be able to explain the presence of repetitive body movements in nearly half (51.2%) of the children included in this study. It should be noted that these repeated body movements, which are known methods of self-regulation in autistic children, can also be a method of managing all modalities of sensory information. From this study, it can also be seen that with the exception of taste, where hyperreactivity is more among children than hyporeactivity, in all the other sensations, including vision, hearing, touch, smell, spatial orientation, and pain, hyporeactivity is a commoner problem than hyperreactivity. The prevalence of hyporeactivity to stimuli in these children indicates that many of these children with sensory hyporeactivity will engage in sensory-seeking behaviors, such as seeking out brightly colored objects, standing close to sources of sound, and constantly running, jumping, and hopping, by which they look for more sensory stimulation. If these behavioral problems are to be corrected by therapy, the underlying motivation, the sensory hyporeactivity, should be understood. The presence of these sensory issues demands to be addressed, as it can disrupt the process of therapy, thereby hindering the child from showing improvement. For the therapy to show progress, any sensory issues a child may have should be identified first, so that appropriate interventions can be carried out to reduce them.

When considering the behavioral issues, 91.9% of the children were found to have some form of behavioral issue, with 81.3% having stereotyped behaviors. This is much higher than the results obtained from a previous study in Sri Lanka [6], which states that only 38.0% of the children have behavioral issues, including stereotyped behavior. The study in Italy [19] however, reported a similar percentage (78.1%) of children with stereotyped behaviors. Previous studies in Sri Lanka have revealed hyperactivity to be a presenting complaint in 4.9% of the children [6], and more recently, 8.0% [18]. However, this study found that of all the 123 children who had been brought Mathavam, 37.4% had hyperactivity as one of the presenting complaints. Aggressive behavior was reported in 13.0% of the children, which is slightly less than the 16% reported in a previous Sri Lankan study [18].

The other major complaint was poor social interaction, which was reported in a vast majority of the children (96.7%). A European study also reported a similarly high percentage of 93.3% [19], even though it did not specify which aspect of social interaction most children were having difficulty with.

Early intervention is known to have a favorable effect on the recovery of autistic children [21]. However, the effectiveness of the intervention can be hampered by certain challenges faced by the therapists, as well as the parents. This study found that parents had trouble managing the demands of therapy as well as caring for the autistic child due to financial hardships, lack of transport facilities, poor family support, and the need to divide their time to take care of other children and other members of the family. Above all, social stigma also hindered their commitment to therapy. Social stigma related to autism, especially in the form of judgment from others and refusal for school admissions, has been reported in a study among parents of autistic children in Ratnapura, a district in the southern part of Sri Lanka [22]. This study, which explored the parental perspectives on the lived experience of having an autistic child, further states the financial burden, limited access to health and educational services, caregiver burden, emotional stress, and dealing with challenging behaviors are among the key challenges faced by these parents.

On the side of therapists, they perceived their efficiency to be hampered by undue pressure exerted on them by the unrealistic expectations of the parents, as well as the demanding nature of some parents. Further, since parents function as co-therapists, the parents’ lack of understanding of the therapies, or their inability to comply with the therapists’ instructions can be detrimental to the recovery of the child. Poor attendance to the therapy sessions due to various reasons including, but not limited to, financial issues, and transportation-related issues, as found from this study, can also be detrimental to the child’s progress. Most of these challenges can be overcome by improving the communication between the therapists and parents, and by enabling further training and qualification of the therapists.

Like any study, this study also has its limitations. Since data were extracted from existing records, a thorough exploration into the possible etiology and risk factors was not possible. Further, the lack of exhaustive notes on the therapy sessions and the challenges encountered hinders the detailed analysis of the challenges faced by the parents and the therapists at this center.

## Conclusions

This study illustrates the current trend of the clinical presentation of ASD in the region, in which all the children present with language problems, while poor social interaction and behavioral problems are found in most of the children. In addition, more than three-fourths of the children had some form of sensory issue. Thus, these findings indicate the necessity to focus on behavioral interventions, speech and language therapy, and occupational therapy and physiotherapy for sensory integration. They also indicate that the sensory issues of the children should be identified and intervened in order to gain maximum benefit from the therapies. This study further explores the challenges faced by the service providers and service recipients, thereby pointing out the need to devise strategies to overcome these challenges.

The findings of the study also highlight the importance of further strengthening the maternal and child care system in Sri Lanka to gain more factual awareness regarding autism in order to sensitize the parents, so that they can identify the red flag signs early and seek appropriate professional help. In addition, advocacy is needed at all levels to bring attention to the magnitude of the need and invest more in developing services for autism and related conditions.

## References

[1] Zeidan J, Fombonne E, Scorah J (2022). Global prevalence of autism: a systematic review update. Autism Res.

[2] Perera H, Wijewardena K, Aluthwelage R (2009). Screening of 18-24-month-old children for autism in a semi-urban community in Sri Lanka. J Trop Pediatr.

[3] Hossain MD, Ahmed HU, Jalal Uddin MM (2017). Autism spectrum disorders (ASD) in South Asia: a systematic review. BMC Psychiatry.

[4] (2019). Jaffna District Secretariat. Annual Performance and Accounts Report.

[5] Chlebowski C, Green JA, Barton ML, Fein D (2010). Using the childhood autism rating scale to diagnose autism spectrum disorders. J Autism Dev Disord.

[6] Perera H, Jeewandara KC, Guruge C, Seneviratne S (2013). Presenting symptoms of autism in Sri Lanka: analysis of a clinical cohort. Sri Lanka J Child Health.

[7] Loomes R, Hull L, Mandy WP (2017). What is the male-to-female ratio in autism spectrum disorder? A systematic review and meta-analysis. J Am Acad Child Adolesc Psychiatry.

[8] Bhuiyan MR, Islam MZ, Rafi A, Al Kawsar A, Akhtar K (2017). Socio-demographic characteristics and related factors affecting children with autism spectrum disorder. J Armed Forces Med Coll Bangladesh.

[9] DiGuiseppi CG, Daniels JL, Fallin DM (2016). Demographic profile of families and children in the Study to Explore Early Development (SEED): case-control study of autism spectrum disorder. Disabil Health J.

[10] Sathyabama R (2019). Clinical characteristics and demographic profile of children with Autism Spectrum Disorder (ASD) at child development clinic (CDC), Penang Hospital, Malaysia. Med J Malaysia.

[11] Hussein H, Taha GR, Almanasef A (2011). Characteristics of autism spectrum disorders in a sample of Egyptian and Saudi patients: transcultural cross sectional study. Child Adolesc Psychiatry Ment Health.

[12] (2022). The World Bank. Health Indicators Accessed. https://data.worldbank.org/topic/health?view=chart.

[13] Bhat BA, Hussain AM, Qadir W, Dar SA (2019). Clinico-socio demographic profile of children with autism spectrum disorders from a tertiary care hospital in Kashmir, India. J Child Dev Disord.

[14] Elder JH, Kreider CM, Brasher SN, Ansell M (2017). Clinical impact of early diagnosis of autism on the prognosis and parent-child relationships. Psychol Res Behav Manag.

[15] Priyatharsan K, Sansiya T, Sinthuja R (2021). Knowledge on autism among mothers of Kopay Medical Officer of Health area during their first year of motherhood. Proc 4th Undergrad Res Symp.

[16] Sriskantharajah L, Thabotharan D, Kamalaruban L, Sathiadas MG (2020). Knowledge and attitude on autism spectrum disorder among public health midwives of Jaffna district. Proc 3rd Undergrad Res Symp.

[17] Begum FA, Rahman AS, Islam MS (2019). Socio-demographic status of children with autism spectrum disorder and their parents in Dhaka city. Int J Hum Health Sci.

[18] Ginige P, Wijesinghe K, Tennakoon S, Alahakoon H, Baminiwatta A (2018). Autism presenting to two child mental health clinics at a tertiary care center in central Sri Lanka: a retrospective clinical record review. Indian J Soc Psychiatry.

[19] Parmeggiani A, Corinaldesi A, Posar A (2019). Early features of autism spectrum disorder: a cross-sectional study. Ital J Pediatr.

[20] Chang YS, Owen JP, Desai SS (2014). Autism and sensory processing disorders: shared white matter disruption in sensory pathways but divergent connectivity in social-emotional pathways. PLoS One.

[21] Helt M, Kelley E, Kinsbourne M, Pandey J, Boorstein H, Herbert M, Fein D (2008). Can children with autism recover? If so, how?. Neuropsychol Rev.

[22] Mahagamage BA, Rathnayake LC, Chandradasa M (2021). Parental perspectives on the lived experience of having a child with autism spectrum disorder in Sri Lanka. Sri Lanka J Child Health.

